# Influence of dual task and frailty on gait parameters of older community-dwelling individuals

**DOI:** 10.1590/bjpt-rbf.2014.0034

**Published:** 2014

**Authors:** Rita C. Guedes, Rosângela C. Dias, Leani S. M. Pereira, Sílvia L. A. Silva, Lygia P. Lustosa, João M. D. Dias

**Affiliations:** 1 Programa de Pós-Graduação em Ciências da Reabilitação, Universidade Federal de Minas Gerais (UFMG), Belo Horizonte, MG, Brazil; 2 Departamento de Fisioterapia, Universidade Federal de Alfenas (UNIFAL), Alfenas, MG, Brazil

**Keywords:** aged, frail elderly, dual task, gait parameters, gait speed, physical therapy

## Abstract

**Background:**

Gait parameters such as gait speed (GS) are important indicators of functional capacity. Frailty Syndrome is closely related to GS and is also capable of predicting adverse outcomes. The cognitive demand of gait control is usually explored with dual-task (DT) methodology.

**Objective:**

To investigate the effect of DT and frailty on the spatio-temporal parameters of gait in older people and identify which variables relate to GS.

**Method:**

The presence of frailty was verified by Fried's Frailty Criteria. Cognitive function was evaluated with the Mini-Mental State Exam (MMSE) and gait parameters were analyzed through the GAITRite^(r)^ system in the single-task and DT conditions. The Kolmogorov-Smirnov, ANOVA, and Pearson's Correlation tests were administered.

**Results:**

The participants were assigned to the groups frail (FG), pre-frail (PFG), and non-frail (NFG). During the DT, the three groups showed a decrease in GS, cadence, and stride length and an increase in stride time (p<0.001). The reduction in the GS of the FG during the DT showed a positive correlation with the MMSE scores (r=730; p=0.001) and with grip strength (r=681; p=0.001).

**Conclusions:**

Gait parameters are more affected by the DT, especially in the frail older subjects. The reduction in GS in the FG is associated with lower grip strength and lower scores in the MMSE. The GS was able to discriminate the older adults in the three levels of frailty, being an important measure of the functional capacity in this population.

## Introduction

Walking is a complex functional activity influenced by several factors such as the subject's health status, motor control, musculoskeletal condition, sensory and perceptual function, level of habitual activity, as well as their environmental characteristics^1^. Gait parameters are important indicators of functional capacity^2^^,^ 3, with especial emphasis on gait speed (GS), as it is considered a reliable, valid, sensitive, and specific measure^4^, besides being simple, quick, and easily administered, both in outpatient and domiciliary environments^5^. GS can be used as an indicator of physiological reserve and it is able to predict falls, frailty, institutionalization, and death among elderly individuals^5^^-^ 8. Its predictive capacity is due to the integration of multiple domains such as the natural ageing process, physical capacity, and the subject's nutritional and emotional status^2^^,^ 9. According to Studenski et al.^2^, GS can be considered the sixth vital sign, because it reflects hidden pathological problems and predicts important future events^2^. The capacity to develop an independent, safe, and fast gait is crucial to good functional performance in human beings^10^.

Locomotion or gait requires adaptive skills to meet individual and environmental demands and often involves the simultaneous performance of cognitive tasks associated with gait, such as recalling a shopping list or having a conversation^11^. Thus, gait is a task that requires attention^12^^-^ 14. The cognitive demand of gait control can be explored with the dual-task (DT) methodology, where performance changes in either or both of the concurrent tasks indicate the extent of their cognitive demand^15^. The hypothesis is that the two tasks interfere with each other and compete for attention resources^15^^,^ 16. DT is clinically relevant because most activities of daily living (ADLs) require the simultaneous performance of two or more tasks, which makes this methodology representative of actual daily situations. Moreover, it constitutes a simple, non-invasive method that does not require specific equipment for its use in clinical practice^17^. For example, a spontaneous narrative is a complex cognitive task: to answer a question, a person must retrieve information from memory, identify the words to encode these meanings, compute the proper grammatical forms, and translate them into motor commands to articulate words^15^. Gait alterations associated with the aging process have been interpreted as a more cautious gait pattern, adopted to increase stability and reduce the risk of falls. However, a more conscientious pattern may require higher cognitive control and result in the need for higher attention to locomotion, making the elderly gait more sensitive to DT^18^. The risk of falls increases as the number of predisposing factors grows^19^. Among these factors, frailty is the most relevant^20^.

Frailty Syndrome (FS) is closely related to GS and is also capable of predicting adverse outcomes such as disability, hospitalization, institutionalization, falls, and death^21^^-^ 23. In spite of the current lack of a consensus on its definition, it has been stated that it is a clinical syndrome of multifactorial nature, characterized by a state of physiological vulnerability resulting from the reduction in energy reserves and in the ability to maintain or restore homeostasis to cope with stressors^24^. Fried et al.^25^ proposed five criteria to identify FS, namely unintentional weight loss in the previous year, muscle weakness, gait slowness, low levels of physical activity, and the feeling of exhaustion. Recent studies suggest that GS has a close correlation with FS and with future adverse events, therefore constituting a practical and reproducible method of diagnosis that is able to identify the frail elderly^26^^,^ 27.

It is known that both frailty and DT lead to changes in gait. However, it is necessary to identify whether DT affects spatio-temporal parameters of gait differently in older people at different levels of frailty. The hypothesis of this study is that DT has a prominent influence on the gait of frail elderly subjects. A better understanding of the interaction between DT, frailty, and gait could help researchers and professionals to plan appropriate intervention studies and help clinicians in the decision-making process. In particular, this study aims to investigate the effect of DT and frailty on the spatio-temporal parameters of gait in older people as well as to identify which variables relate to GS at the different levels of frailty (cognitive function, handgrip strength, and number of diseases).

## Method

### Sample

Eighty-one individuals of both genders, selected by convenience, participated in the study. The exclusion criteria were: surgical procedure in the lower limbs or in the vertebral column in the last year; reported pain in the lower limbs on the day of the assessment or inability to walk without a walking aid for one minute; severe balance impairment; uncompensated neurological, cardiac or vascular conditions; musculoskeletal diseases that could hinder the performance of the tests; and a clinical scenario suggestive of cognitive alterations ascertained by the Mini-Mental State Exam (MMSE)^28^. The participants signed an informed consent form agreeing to participate. This study was approved by the Research Ethics Committee of Universidade Federal de Minas Gerais (UFMG), Belo Horizonte, MG, Brazil (protocol no. CAAE-0700.0.203.000-11).

### Instruments

The presence of frailty was verified by the five components of Fried's frailty criteria^25^: unintentional weight loss in the last year (≥4.5 kg or 10% of body weight); self-reported exhaustion (determined by the answers "a moderate amount of the time" or "most of the time" to one of the two statements - "I felt that everything I did was an effort" and "I could not get going"); diminished hand grip strength (measured with a hand grip dynamometer - JAMAR) with cutoff points determined by the calculation of the 20^th^ percentile of the sample, adjusted for gender and body mass index; gait slowness (determined by the time spent in seconds to cover a distance of 4.6 m at a comfortable speed, also with cutoff points defined at the 20^th^ percentile of the sample adjusted for gender and height); and low level of physical activity (determined using the Active Australia^29^ questionnaire with cutoff points determined by the 20^th^ percentile of the sample for men and women).

This questionnaire is used in population surveys to determine the weekly caloric expenditure and contains information about frequency, intensity, duration, and type of activity. It is a reliable instrument that can be applied quickly and is valid for use in community-dwelling elderly^30^. Elderly adults with a positive score in 3 or more of the 5 criteria were considered frail; those with one or two positive items, pre-frail; and those with all negative scores were considered non-frail.

To screen for possible cognitive deficits, the MMSE was administered. This instrument is comprised of seven categories, each of them designed to assess temporal and spatial orientation, naming and subsequent recalling of three words, attention and calculation, language and visual constructive praxis. The MMSE score ranges from zero to 30 points, and the following cutoff points were considered according to the level of education: 13 for illiterate, 18 for one to seven years of schooling, and 26 for those with eight or more years of schooling^28^.

The gait parameters were analyzed using the GAITRite^(r)^ system (MAP/CIR INK, Haverton, PA, USA), which consists of an electronic vinyl carpet capable of registering the plantar impression, allowing the calculation of spatial and temporal gait data^27^. The carpet is 90 cm wide by 566 cm long by 0.6 cm high and contains 18,824 embedded pressure sensors. The system has software for data analysis and documentation of nine temporal parameters and six spatial parameters. The present study used the data from GS, cadence (CAD), stride length (SL), and stride time (STi). A number of studies have shown the validity and reliability of its measures compared to existing techniques, including studies in older adults^31^^-^ 33.

### Procedures

Initially, the participants responded to a questionnaire with demographic and clinical data created for this study to characterize the participants and classify them as frail, pre-frail, and non-frail. After that, the MMSE was administered to screen for possible cognitive deficits. The gait analysis on the GAITRite^(r)^ system was performed at two time points. First, the participants were asked to walk in silence for 1 minute on the carpet, characterizing the single task (ST), then after a 5-minute interval, they were asked to walk for 1 more minute on the carpet while responding to the question "What was the best moment of your life and why?", characterizing the DT. If the participant finished answering before the end of the 1 minute, the researcher asked a new question regarding the theme to keep the participant talking for the duration of the assessment.

### Statistical analysis

Descriptive analysis was done using mean and standard deviation for continuous variables. The Kolmogorov-Smirnov test determined the normal distribution of data, justifying the use of parametric tests. To evaluate differences between non-frail, pre frail, and frail older people in relation to age, body mass index, handgrip strength, MMSE, number of diseases and number of medicines, we used ANOVA with Tukey's post-hoc test. In the comparisons of gait parameters (GS, CAD, SL, and STi) in ST and DT situations, a 3×2 repeated-measures ANOVA (three levels of frailty × two tasks) was used. Correlations between VM, age, BMI, handgrip strength, MMSE, number of diseases and medications were investigated using Pearson's test. The level of significance was set at 5% for all the tests. The power was set at 80% to detect differences between the variables.

## Results

The participants included in the study were assigned to the groups frail (FG), pre-frail (PFG), and non-frail (NFG). The clinical and demographic data (Table 1) show that the FG is composed of participants with a higher number of diseases who used a higher number of medications regularly, in addition to presenting lower handgrip strength and lower scores in the MMSE.

**Table 1:** Demographic and clinical characteristics of the participants (n=81).

Variables	Frail Group (n=27)	Pre-Frail Group (n=27)	Non-frail Group (N=27)
Age (years)	75.48±7.08*	70.11±7.30	69.6±5.45
Gender (female / male)	f=21; m=6	f=22; m=5	f=20; m=7
BMI (Kg/m2)	27.64±7.11	27.02±5.57	26.34±4.56
Height (m)	1.56±0.30	1.56±0.96	1.58±0.88
Handgrip strength (Kgf)	20.53±5.25*	25.83±6.68	27.25±7.38
MMSE (score)	22.44±4.73*	27.12±2.71	27.41±1.14
Diseases (number)	6.82±1.35*	2.5±1.46	2.4±1.25
Medicine (number)	8.41±4.17*	3.48±2.34	2.96±2.02

BMI = body mass index

Kgf = kilogram force

MMSE = Mini-Mental State Exam

The values were described as mean and standard deviation, except for gender

* p≤0.05 for between-group comparison (one-way ANOVA, Tukey's post hoc).

**Figure 1:**
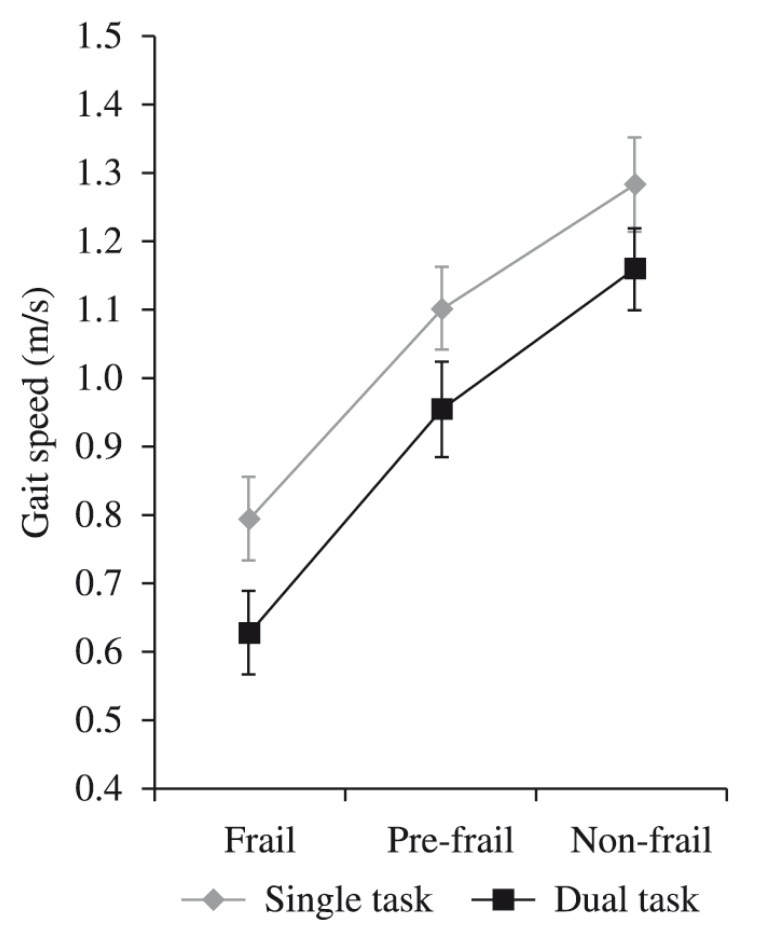
Mean and standard deviation values for gait speed in both tasks (single and dual) for each group (frail, pre-frail, non-frail).

**Figure 2:**
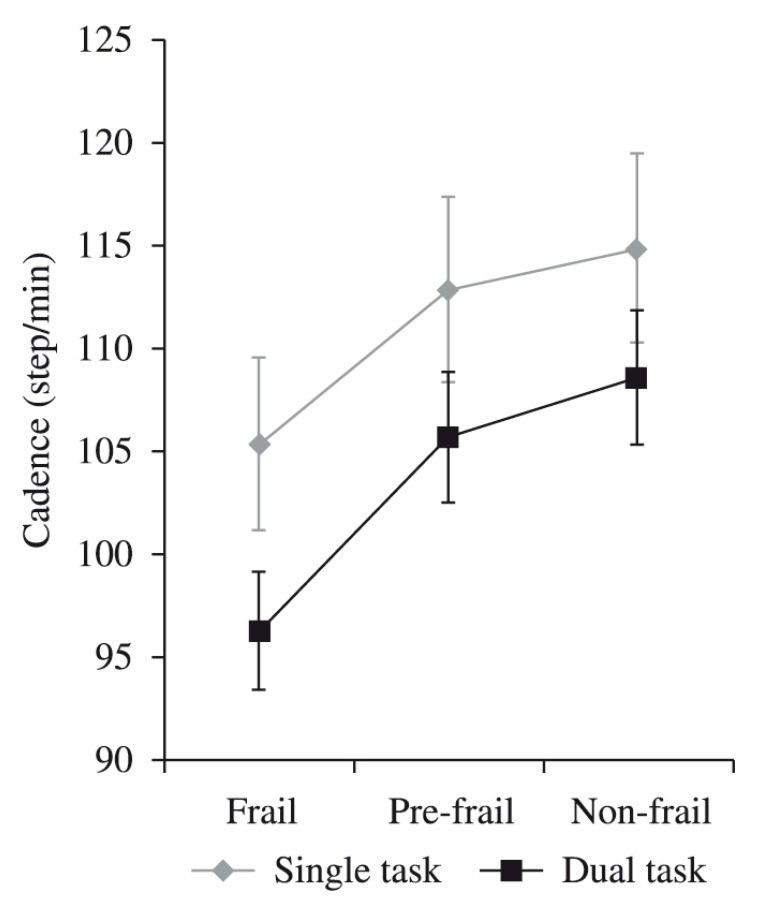
Mean and standard deviation values for cadence in both tasks (single and dual) for each group (frail, pre-frail, non-frail).

**Figure 3:**
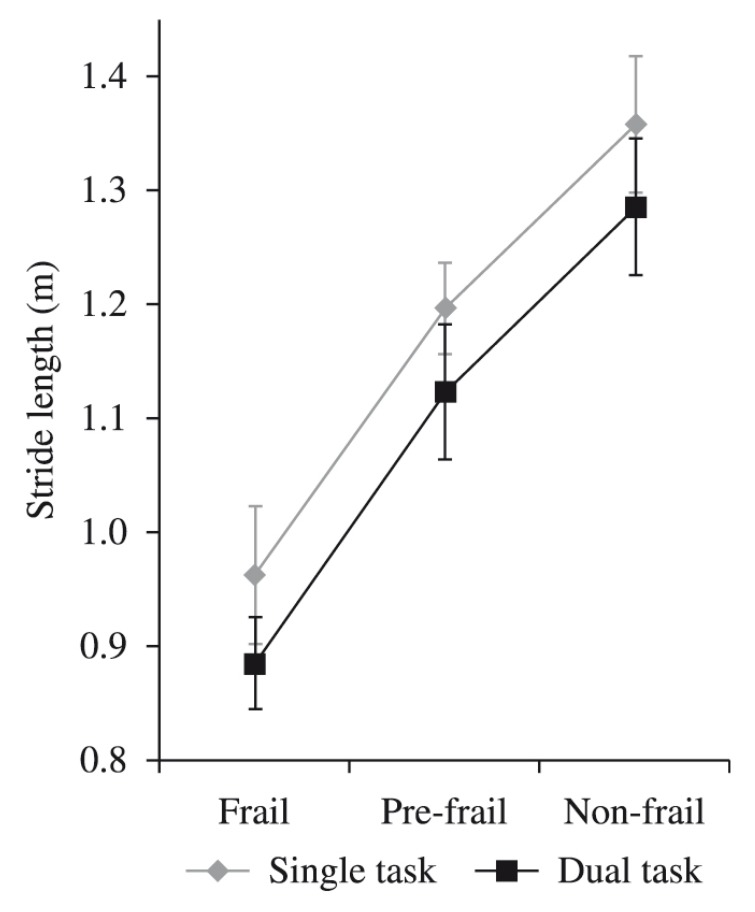
Mean and standard deviation values for stride length in both tasks (single and dual) for each group (frail, pre-frail, non-frail).

Figures 1, 2, and 3 showed the effect of the DT in the three groups, leading to statistically significant reductions in GS (m/s), CAD (steps/min), and SL (cm), respectively. Repeated-measures ANOVA showed effects of group (F2,52=226.57, p=0.000) and task (F1,26=447.59, p=0.000), but not of interaction in GS (F2,52=3.03, p=0.057). Regarding CAD, this test showed effects of group (F2,52=9.65, p=0.001) and task (F1,26=40.84, p=0.000), but not interaction (F2,52=0.60, p=0.512). Concerning SL, this statistical test also showed effects of group (F2,52=73.53, p=0.000) and task (F1,26=117.82, p=0.000), but not interaction (F2,52=0.046, p=0.955).

In the DT situation, the GS fell by 20% in the FG, 13.2% in the PFG, and 10% in the NFG compared to the ST situation. CAD fell by 8.6% in the FG, 6.4% in the PFG, and 5.5% in the NFG. SL fell by 8% in the FG, 6.1% in the PFG, and 5.3% in the NFG. In relation to STi (s), there was an increase of 15.2% in the GF, 6.4% in the PFG, and 5% in the NFG compared to the ST situation.

Table 2 shows that, in the ST and DT situations, GS and SL were different in the three groups (FG<PFG<NFG). It can also be observed that both in the ST and DT situations, the FG differed to the other groups with regards to the spatio-temporal gait parameters.

**Table 2:** Spatio-temporal parameters in the single task and dual task situations tested (n=81).

Variables	Frail Group (n=27)	Pre-Frail Group (n=27)	Non-Frail Group (n=27)
Gait speed ST (m/s)	0.79±0.13	1.10±0.0 *	1.28±0.07 **¥ ** **¤ **
Cadence ST (steps/min)	105.36±13.39	112.87±7.10*	114.90±6.40 **¥ **
Stride time ST (s)	1.18±0.14* **¥ **	1.08±0.74	1.05±0.56
Stride length ST (m)	0.96±0.16	1.19±0.10*	1.35±0.09 **¥ ** **¤ **
Gait speed DT (m/s)	0.62±0.10	0.95±0.08*	1.16±0.06 **¥ ** **¤ **
Cadence DT (steps/min)	96.29±11.94	105.70±9.10*	108.60±13.90 **¥ **
Stride time DT (s)	1.36±0.31* **¥ **	1.15±0.96	1.11±0.05
Stride length DT (m)	0.88±0.15	1.12±0.09*	1.28±0.09 **¥ ** **¤ **

ST = single task

DT = dual task

Values described as mean and standard deviation

* GF x GPF

¥ GF x GNF

¤ GPF x GNF for between-group comparison. (ANOVA with Tukey's multiple comparisons).

The reduction in GS in the FG during the performance of the DT showed a positive correlation with the scores obtained in the MMSE (r=730; p=0.001) and with hand grip strength (r=681; p=0.001). Furthermore, 55.5% (n=15) of the FG and 33.3% (n=9) of the PFG participants were positive for this item of the phenotype for frailty.

## Discussion

The aim of this study was to investigate the effect of DT and frailty on the spatio-temporal parameters of elderly individuals. The results obtained in the present study showed that the impact of spontaneous narrative on the spatio-temporal gait parameters was evident. All of the participants, regardless of their level of frailty, slowed down significantly, with reductions in SL and CAD and increase in STi. The frail participants were the ones with the most intense changes.

Although gait seems to be an automatic motor activity, evidence suggests that the act of walking requires attention to environmental characteristics and the recovery of postural disturbances to avoid falls^12^^-^ 14. The allocation of attention in concurrent activities represents executive processes that are sensitive to the aging process, which makes gait more cautious and more influenced by the DT^34^. The findings of the present study are in accordance with the results obtained in a recent systematic review that highlighted the reductions in GS, CAD, and SL and the increase in STi as the most important changes in gait found in the DT situation in older adults^15^. While approximately 55% of falls are related to abnormal gait^35^ and considering that performing two simultaneous tasks is necessary for independence in ADLs, it becomes necessary to incorporate the DT methodology into the rehabilitation of older persons in general, but especially of frail elderly individuals.

The choice for using the DT methodology was based on the fact that walking and talking simultaneously consists in a very ecological and necessary task to ADLs and seems to require more attention and to produce a higher interference in the motor task^17^^,^ 36. Considering that theoretical framework, Al-Yahya et al.^15^ used the question "what was the best vacation of your life, and why?". In the present study, the same methodology was used, but in order to culturally adapt the meaning of the question, an expert committee was formed to discuss how the question might be modified to adjust to the Brazilian elderly population.

It has been suggested that the size of the interference of the DT on gait is influenced by self-selected GS, with greater changes in subjects with GS≤1.0 m/s^37^^,^ 38 and fewer changes in those with GS≥1.2 m/s^39^. The present study also identified greater changes in the FG (reductions in GS [20%], CAD [8.6%], and SL [8%] and increases in STi [15.2%]) and fewer changes in the NFG (reductions in GS [10%], CAD [5.5%], and SL [5.3%] and increases in STi [5%]). These findings confirm the idea that frail older people walk more slowly and suffer greater influence of DT compared with non-frail older individuals.

If we consider that a GS below 0.6 m/s is associated with dependence in basic and instrumental ADLs and gait limited to the home environment^1^, we can infer that the FG is significantly limited in the performance of motor tasks associated with spontaneous speaking, since this group showed GS=0.60 m/s in the DT situation. In comparison, the GS necessary to safely cross the street at a traffic signal must be equal to or greater than 1.2 m/s^17^^,^ 36 ^,^ 39. The PFG and NFG also had values below this in the DT situation. Considering that walking and talking simultaneously consists in an extremely functional and common action in ADLs, all of the participants would probably have difficulty crossing a street, being therefore at greater risk of accidents and dependence in outdoor mobility.

Epidemiological studies and clinical trials show that gait and cognition are inter-related. Gait changes are associated with falls, dementia, and disability^12^^,^ 13, and gait speed reduction can start up to 12 years before the clinical presentation of cognitive impairment^13^. Moreover, changes in attention, memory, and executive function are related to gait slowness and help to predict loss of mobility, falls, and progression of cognitive decline^12^. There is robust evidence to suggest a strong correlation between cognitive level measured by the MMSE and GS, and this relationship becomes more evident when the task is more challenging or when the gait pattern is already impaired^12^^,^ 13 ^,^ 40 ^-^ 42. In the current study, this association was identified only in the FG, reinforcing the hypothesis that lower scores in the MMSE can reduce the allocation sources for attention, compromising gait.

Considering that GS is the product of CAD and SL^43^, one can observe that frail, pre-frail, and non-frail older adults use the same gait adaptation strategies in the DT situation; thus, they reduce CAD and SL, with a consequent reduction in GS. The FG was the group that showed a more accentuated reduction in GS during the DT, and this reduction showed a strong positive correlation with handgrip strength. It is known that this measure is able to represent global strength, and that lower values are related to sarcopenia^44^. The lower muscle strength of the FG may have played a significant role in GS reduction, impairing gait propulsion and consequently reducing SL.

During the last years, GS has been reported as an efficient measure to identify older adults at higher risk of adverse events, as it is an easy, simple, and low-cost measurement that can be used both in clinical settings and research^45^. In the present study, GS was able to differentiate the three groups, both in the ST and DT situations. The participants from the FG showed lower GS and more chronic diseases, used a greater number of medications, had lower handgrip strength, and showed lower cognitive ability. Similarly to Rothman et al., more than half of the GF and a third of the PFG participants scored positively on the GS item of the frailty criteria^46^. These findings allow us to deduce that GS plays an important role in the Frailty Syndrome classification and, additionally, might provide information about the general health status of older individuals, being thus an important vital sign measure for functional capacity in this population.

Despite the statistically significant negative correlation found between GS and MMSE scores due to the exclusion criteria of the study, it was not feasible to analyze this correlation for individuals with cognitive deficits ascertained by the MMSE scores. Thus, future research must address this issue.

## Conclusion

The results have shown that the gait of frail older adults is more affected by the dual task, showing a greater reduction in speed, cadence, and stride length and increase in stride time compared to pre-frail and non-frail older adults. The reduction in gait speed in the frail elderly is associated with lower hand grip strength and lower scores in the MMSE. Moreover, gait speed was able to discriminate the older subjects, stratifying them into the three levels of the frailty syndrome, thus being an important measure of functional capacity in this population. Considering the importance of DT in the ADLs of older individuals, this methodology should be part of a comprehensive functional assessment and physical therapy approach designed for these individuals, particularly those who are frail with lower MMSE scores and handgrip impairment.
